# COVID-19 in Cirrhotic Patients: Is Portal Vein Thrombosis a Potential Complication?

**DOI:** 10.1155/2022/5900468

**Published:** 2022-03-26

**Authors:** Alshymaa A Hassnine, Amr M Elsayed

**Affiliations:** Department of Tropical Medicine, Faculty of Medicine, Minia University, Minia, Egypt

## Abstract

**Introduction:**

Several studies have demonstrated that thromboembolic events increased in patients with coronavirus infection, usually occurring in elderly patients with severe illness, associated with comorbid diseases such as diabetes and hypertension. Portal vein thrombosis (PVT) is a rare venous thromboembolic disease occurring typically in patients with an underlying disease such as decompensated cirrhosis with or without hepatocellular carcinoma (HCC).

**Aim:**

To evaluate incidence of occurrence of acute PVT in cirrhotic patients infected with 2019 coronavirus disease (COVID-19).

**Methods:**

This cross-sectional, observational study involved 70 patients of the liver cirrhosis: (group A) 28 patients with liver cirrhosis infected with COVID-19, and 42 patients with liver cirrhosis as the control group matched for age and sex (group B). All patients were subjected to thorough medical history, routine investigations (complete blood count, liver, and renal function tests), imaging in the form of abdominal and Doppler ultrasonography to assess the presence of acute PVT, serum ferritin, D-dimer, C-reactive protein, and PCR of COVID-19 for group A only.

**Results:**

There was a significant difference between the two groups regarding Doppler ultrasound findings as 3 of the patients in group A had PVT (10.7%), 2 of them had HCC diagnosed by triphasic CT abdomen, and only 1 patient in group B had PVT (2.3%) (*p* < 0.05).

**Conclusion:**

In cirrhotic patients infected with COVID-19, portal vein thrombosis may be a potential complication even in the absence of hepatocellular carcinoma; further prospective studies with longer follow-up may be needed.

## 1. Introduction

A new severe acute respiratory syndrome coronavirus began in Wuhan in December 2019 and then rapidly spread throughout China and became worldwide [1]. With time, some new extrapulmonary manifestations of this viral pneumonia were described. Increased incidence of thromboembolic events was frequently reported [2], and multiple studies showed that thromboembolic events increased in patients with coronavirus infection, usually occurring in the elderly patients with severe illness, associated with comorbid diseases such as diabetes and hypertension [2].

Portal vein thrombosis is a rare venous thromboembolic disease typically occurring in patients with an underlying disease such as decompensated liver cirrhosis with or without hepatocellular carcinoma, systemic lupus erythematous, pancreatitis, and other hypercoagulable conditions [3, 4]. Several studies showed that thromboembolic events increased in patients with coronavirus infection since the pandemic declared by the World Health Organization [5, 6]; in these studies, thromboembolic events such as deep venous thrombosis, cerebral infarction, and pulmonary emboli usually were occurring in the elderly patients with severe COVID-19 associated with comorbid diseases such as diabetes and hypertension [7–9]. PVT is one of the most common vascular disorders of the liver with significant morbidity and mortality [10]. A global prevalence of 1% have been reported in large cohort studies, but it may be up to 26% in some risk groups [11, 12]. Causes of PVT are liver cirrhosis, hepatobiliary malignancy, abdominal infectious or inflammatory diseases, and myeloproliferative disorders [13]. Most patients with PVT have a general or local risk factor [12]. The natural history of PVT leads to portal hypertension, splenomegaly, and the formation of portosystemic collaterals, esophageal, and or gastric varices [14].

The diagnosis of PVT in patients with cirrhosis is usually made during routine ultrasound examination in asymptomatic patients or following a new event of hepatic decompensation [15]. Splanchnic Doppler ultrasound is the first-line method used to detect PVT in cirrhotic patients with a sensitivity of about 90% for complete thrombus, which decreases to about 50% for partial thrombosis. Additionally, a CT scan or MRI can better define the extension of PVT [16].

## 2. Subjects and Methods

This cross-sectional, observational control study involved 70 patients of liver cirrhosis with or without decompensation divided into two groups: group A (28 patients with liver cirrhosis infected with COVID-19), and 42 patients with liver cirrhosis only as the control group matched for age and sex (group B). The patients with liver cirrhosis were selected randomly from the outpatient clinic of the Tropical Medicine department, Minia University Hospital, during the period from January 2021 to March 2021. Patients were collected according to the following inclusion criteria: cirrhotic patients presented with acute onset of fever, any upper respiratory symptoms, history of fatigue, acute abdominal pain, and/or history of diarrhea and vomiting; patients were excluded from the study if they had any history of PVT or any other thromboembolic events before the symptoms of the COVID-19 has appeared. Informed consent was obtained from all subjects (for both participation and publication of that work). The local ethics committee for human subject research reviewed and approved the study protocol and consent forms; all procedures performed in the study were in accordance with the ethical standards of the national research committee.

All patients were subjected as follows:

1. History taking including the personal history, present illness of acute onset of fever, any upper respiratory symptoms, history of fatigue, acute abdominal pain, history of diarrhea and vomiting, and history stressing on history of DM
2. Basic investigations including complete blood count (CBC), liver and renal function tests, and INR
3. Imaging in the form of abdominal and Doppler ultrasonography to assess the presence of PVT
4. Serum ferritin, D-dimer, C-reactive protein, LDH, and PCR of COVID-19 for group A patients only

The collected data were inserted, tabulated, and statistically anatomized using software version 24 of Statistical Package for Social Sciences program (SPSS). Quantitative data were expressed as mean + standard deviation (SD), while qualitative data were expressed as proportions. Comparisons between groups for qualitative data were analyzed by test of proportion of the *Z* test between the two groups. Statistical significance was defined as *p* values less than 0.05.

## 3. Result

**Figure 1:**
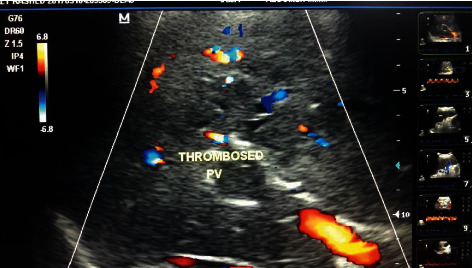
Doppler ultrasound examination of PVT.

Demographic and laboratory characters of the studied cases are given in Table 1; there was no significant difference between the two groups regard age, sex, child, and meld scores, in group A, 8 of the patients had history of DM (29%), and in group B, 13 patients were diabetic (31%). There was mild elevation in liver enzymes in group A than in group B with no statistically significant difference between two groups regarding ALT, AST, albumin, INR, creatinine, and bilirubin level. Lymphocytic count was significantly lower in group A (13%) than in group B (21%). In group A, 7 of the patients had HCC (25%), according to abdominal computerized tomography findings, and in group B, 9 patients had HCC (21.5%). There was a significant difference between the two groups regarding Doppler ultrasound findings as 3 of the patients in group A had PVT (10.7%), 2 of them had HCC diagnosed by triphasic CT abdomen, and only 1 patient had PVT in group B (2.3%). Clinical presentation of the group A is given in Table 2; the most common symptom is fatigue which was present in 23 patients (82.1%), fever which was present in 22 of the patients (78.5%), abdominal pain was present only in 13 patients (46.4%), and most of the patients presenting with chest symptoms in the form of cough (64.2%) and dyspnea (71.4%). Only 9 patients had vomiting, and 12 patients were presented by diarrhea. Laboratory parameters of group A are given in Table 3. Doppler ultrasound examination of PVT is shown in Figure 1.

## 4. Discussion

Portal vein thrombosis is considered the commonest thrombotic complication in patients with hepatocellular carcinoma on top of liver cirrhosis [17]. PVT is a venous thromboembolic disease occurring typically in patients with an underlying disease such as decompensated cirrhosis with or without HCC and other hypercoagulable states [18]. It is still debatable if SARS-CoV-2 has a direct effect on hepatocytes or on liver parenchyma or not [19]. It has been observed that metabolic associated fatty liver disease (MAFLD) may be associated with a relatively higher risk of severe COVID-19 [20]. Multiple studies show that thromboembolic problems such as pulmonary emboli, deep venous thrombosis, and cerebral infarction typically occur in the elderly patients with severe COVID-19 and comorbid diseases such as hypertension and diabetes [6]. We report a coincidence of the acute portal vein thrombosis and COVID-19 respiratory disease in patients of liver cirrhosis, as we found that portal vein thrombosis occurred in about three patients (10.7%) of cirrhotic patients with COVID-19 not previously known to have portal vein thrombosis versus only one patient (2.3%) in non-COVID-19 cirrhotic patients with a significant *p* value (˂0.05). The three COVID-19 patients were diabetic, two of them had HCC diagnosed by triphasic CT abdomen. Many case reports described the occurrence of acute portal vein thrombosis as a consequence of COVID-19 infection in noncirrhotic patients, and the condition was associated with ground glass opacities in CT chest [21–24]. Other case reports mentioned the association of portal vein thrombosis with COVID-19 without any chest manifestations [8, 25, 26]. Thus, in summary, we conclude that in cirrhotic patients with COVID-19 infection, portal vein thrombosis may be a potential complication even in the absence of hepatocellular carcinoma. The main limitations of our study arise from relatively small number of the patients, and the long-term effect of the COVID-19 on LC is not completely established, and further prospective studies with a larger scale study and longer follow-up may be needed for more demonstration of this interesting finding and to establish the pathogenic mechanisms of portal vein thrombosis in cirrhotic patients infected with COVID-19.

## Figures and Tables

**Table 1:** Demographic and laboratory characters of the studied group.

	Group A (28)	Group B (42)	*P* value
Age (median in years)	46 (17–71)	48 (21–66)	0.786
Female/male	11/17	17/25	0.543
Child–Pugh classification [27] (number and %)			0.098
A	5 (18%)	7 (17%)	
B	16 (57%)	26 (62%)	
C	7 (25%)	9 (21%)	
Diabetes mellitus	8 (29%)	13 (31%)	0.145
Model for end stage liver disease [28] (MELD), median score	12 (7–34)	11 (8–33)	0.086
INR	1.6 (1.1–2.6)	1.5 (1.2–2.4)	0.465
Bilirubin (mg/dl)	1.5 (1–21)	1.3 (0.9–14)	0.632
Albumin (g/dl)	3 (2.2–4.5)	3.2 (2.4–4.1)	0.076
Creatinine (mg/dl)	1 (0.9–2.8)	0.9 (0.8–6.2)	0.963
ALT (U/L)	44 (18–466)	48 (26–212)	0.065
AST (U/L)	49 (16–512)	52 (22–288)	0.961
Lymphocyte count (%)	13%	21%	0.03
Hepatocellular carcinoma	7 (25%)	9 (21.5%)	0.645
Portal vein thrombosis	3 (10.7%)	1 (2.3%)	0.04

**Table 2:** Clinical presentation of group A.

Symptoms	Number	%
Fever	22	78.5
Cough	18	64.2
Dyspnea	20	71.4
Fatigue	23	82.1
Headache	3	10.7
Abdominal pain	13	46.4
Vomiting	9	32.1
Diarrhea	12	42.8

**Table 3:** Laboratory parameters of group A.

Variables	Level	Median
C-reactive protein (0–5 mg/L)	(1–96)	12
Serum ferritin (16 200 ng/ml)	(20–740)	190
D-dimer (up to 0.5 mg/ml)	(0.1–2.5)	0.5
Lactate dehydrogenase (240–480 unit/l)	(120–490)	185

## Data Availability

The data used to support the findings of this study are included within the article.
